# Demographic Shift of Influenza A(H1N1)pdm09 during and after Pandemic, Rural India

**DOI:** 10.3201/eid1809.111847

**Published:** 2012-09

**Authors:** Shobha Broor, Wayne Sullender, Karen Fowler, Vivek Gupta, Marc-Alain Widdowson, Anand Krishnan, Renu B. Lal

**Affiliations:** All India Institute of Medical Sciences, New Delhi, India (S. Broor, A. Kirshnan);; University of Alabama, Birmingham, Alabama, USA (W. Sullender, K. Fowler);; The Inclen Trust International, New Delhi (V. Gupta);; and Centers for Disease Control and Prevention, Atlanta, Georgia, USA (M.-A. Widdowson, R.B. Lal)

**Keywords:** influenza, seasonal influenza, influenza A(H1N1)pdm09, India, pandemic, viruses, H1N1, pH1N1

## Abstract

Population-based active surveillance in India showed higher incidence rates for influenza A(H1N1)pdm09 among children during pandemic versus postpandemic periods (345 vs. 199/1,000 person-years), whereas adults had higher rates during postpandemic versus pandemic periods (131 vs. 69/1,000 person-years). Demographic shifts as pandemics evolve should be considered in public health response planning.

Influenza epidemics and pandemics has been recognized for centuries (*1*,*2*), and the effects that influenza can have on public health infrastructure were demonstrated globally during the 2009–2010 pandemic (*3*). The dynamics of influenza transmission are dependent on many factors, including probability of infection, susceptible populations within age groups, and close contacts between susceptible and infected persons (*4*,*5*). Data from 3 recent influenza pandemics show that school-aged children have the highest disease rates and may serve as a key source of transmission to adults (*2*,*6*–*8*).

A recent mathematical modeling study suggested that initial exposure to a novel influenza virus among a highly susceptible population (school-aged children) results in a shift in transmission patterns as infection spreads, with adults more affected during later phases (*9*). To investigate if transmission of influenza A(H1N1)pdm09 (pH1N1) followed this demographic shift pattern, we examined comprehensive weekly active community surveillance for febrile acute respiratory illness (ARI) in 3 rural villages in northern India, as well as available extensive epidemiologic data.

## The Study

All residents of 3 villages in Ballabgarh (n = 16,861) in Haryana, India, have been under weekly household surveillance for febrile ARI since November 2009 as part of a clinical trial of seasonal inactivated trivalent influenza vaccine in children 6 months–10 years of age (NCT00934245; www.clinicaltrials.gov); 95% of eligible children were recruited for this trial (Table 1). Information on febrile ARI, which consists of reported fever plus any respiratory complaint (e.g., cough, sore throat, nasal congestion, runny nose, earache, or difficulty breathing), was collected for all household members either by self-report or by proxy by trained field workers. Consent was obtained from all participants.

**Table 1:** Demographic data for persons under surveillance and incidence of febrile ARI and influenza A(H1N1)pdm09 during pandemic and postpandemic periods, Ballabgarh, India*

Demographics and test results	Pandemic period, November 2009–January 2010	Postpandemic period, August–October 2010
Mean no. persons under surveillance (person-years)	7,340 (1,835)	16,396 (4,134)
No. febrile ARI episodes (incidence rate/1,000 person-years)	1,515 (826)	4,933 (1,203)
No. (%) persons tested for influenza	1,094 (72)	3,907 (79)
No. (%) positive for influenza	265 (24)	902 (23)†
No. (%) positive for influenza A(H1N1)pdm09	231 (21)	506 (13)
No. (%) positive for influenza B	34 (3)	377 (10)
Influenza incidence rate/1,000 person-years	205‡	278
Median age, y (interquartile range)		
All persons with influenza	9 (4–17)	15 (6–30)§
Persons with influenza A(H1N1)pdm09	9 (5–18)	18 (7–32)§
Persons with Influenza B	7.5 (4–16)	13 (5–27)

*ARI, acute respiratory infection. †Total no. positive during postpandemic period included 18 persons with influenza A (H3N2) infection and 1 person co-infected with influenza B and A(H1N1)pdm09. ‡The rate of influenza positivity of sampled febrile ARI case-patients was adjusted to unsampled case-patients assuming similar characteristics for the 2 groups. §Wilcoxon rank-sum test, p<0.001.

During November 2009–October 2010, of the 12,896 eligible persons with febrile ARI, samples were collected from 10,002 (78%); missing samples were because those persons were not available for testing at the time of home visit. Throat and nasal swab specimens were collected from all available febrile ARI patients and tested by using real-time reverse transcription PCR (*10*). Incidence rates (IRs; reported as per 1,000 person-years) and corresponding 95% CIs were calculated for the peak periods of influenza circulation. The pandemic period was defined as November 2009–January 2010 and the postpandemic period as August–October 2010 (first postpandemic period). The Institutional Review Boards of All India Institute of Medical Sciences, University of Alabama, and Centers for Disease Control and Prevention approved the study. Informed consent was obtained for all persons included in the study.

**Figure 1:**
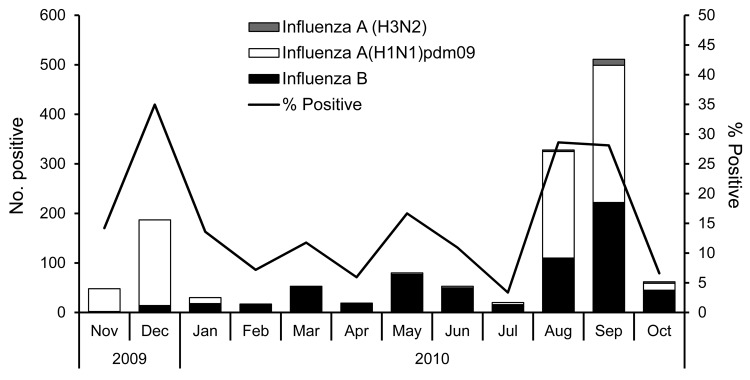
Monthly trends of positive influenza test results during active surveillance in a community-based study, rural India, November 2009–October 2010. Of 1,409 positive test results, 748 (53.1%) were for influenza A(H1N1)pdm09, 642 (45.6%) for influenza B, 18 (1.3%) for influenza A (H3N2), and 1 for co-infection with influenza B and A(H1N1)pdm09. Children 6 months–10 years of age received trivalent seasonal influenza vaccine (intervention) or inactivated polio vaccine (control) during November–December 2009; coverage was 92%.

Two distinct peaks of pH1N1 activity were identified during the pandemic and postpandemic periods (Figure 1), with some circulation during the intervening period (February–July 2010, <0.6%). Rates of positive test results for pH1N1 were higher during peak pandemic (21%) compared with peak postpandemic (13%) periods, whereas influenza B positivity was higher during the postpandemic period (Table 1). The median age of persons with pH1N1 illness during the postpandemic period was significantly higher than during the pandemic period (18 vs. 9 years of age; p<0.001).

**Figure 2:**
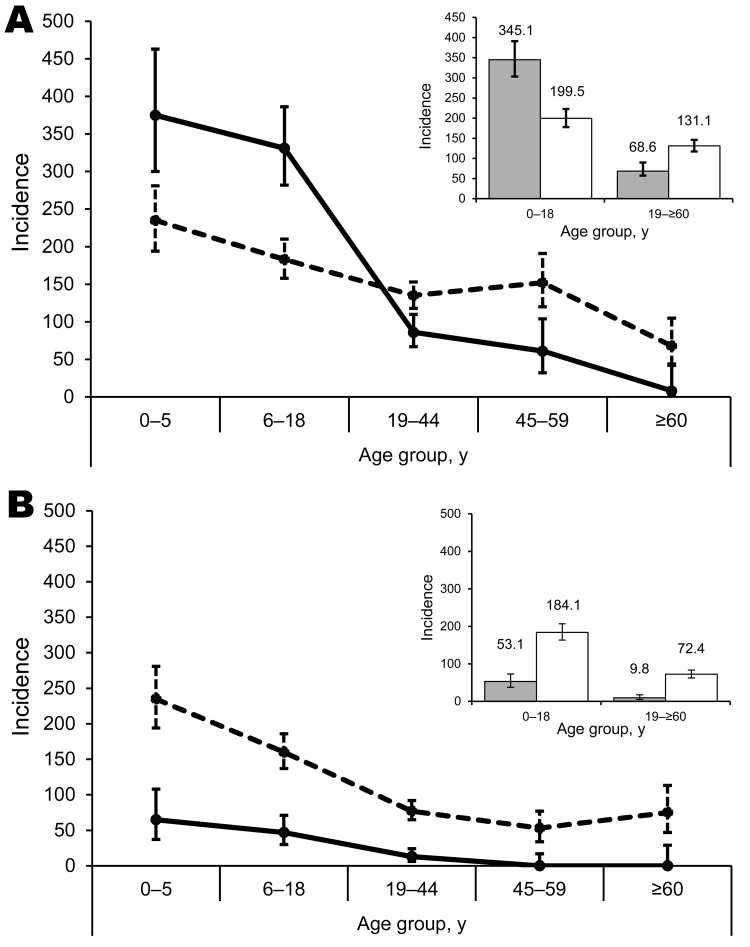
Incidence rates (per 1,000 person-years) for influenza A(H1N1)pdm09 (A) and influenza B (B) during pandemic (November 2009–January 2010; solid lines) and postpandemic (August–October 2010; dashed lines) periods in a rural community in northern India. Cumulative incidence rates for A(H1N1)pdm09 (A, inset) and influenza B (B, inset) during pandemic (gray bars) and postpandemic (white bars) periods are also shown, with incidence rates given on top of the bars. Error bars indicate 95% CIs.

IRs for pH1N1 were higher for children 0–5 and 6–18 years of age (IR 375 and 331, respectively) than for adults (IR 8–86) during the pandemic period (Table 2). The differences in IRs of pH1N1 across age groups disappeared during the postpandemic period, however, this occurred primarily because of a decrease in IRs among the 0- to 5- and 6- to 18-year-old age groups (incidence rate ratio [IRR] 0.6) and concurrent increases among older age groups (IRR 1.6–8.7). These changes were statistically significant (p<0.0001; Figure 2, panel A). The overall IR for influenza B was higher during the postpandemic period; IR for influenza B remained higher for children <18 years of age regardless of pandemic period (Figure 2, panel B).

**Table 2:** Incidence rates for influenza A(H1N1)pdm09 and influenza B among persons with febrile ARI during pandemic and postpandemic periods, by age group, Ballabgarh, India*

Age group, y	Pandemic period, November 2009–January 2010						Postpandemic period, August–October 2010					Incidence rate ratio (95% CI)
	Person-years†	Febrile ARI, no. cases	No. tested‡	No. (%) positive‡	Incidence (95% CI)§		Person-years†	Febrile ARI, no. cases	No. tested‡	No. (%) positive‡	Incidence (95% CI)§	
Influenza A(H1N1)pdm09												
0–5	230	499	411	71 (17.3)	375 (300–463)		498	1,174	1,036	103 (10.0)	235 (194–281)	0.6 (0.5–0.8) ¶
6–18	486	435	281	104 (37.0)	331 (282–386)		1,061	1,245	1,012	158 (15.6)	183 (158–210)	0.6 (0.4–0.7)¶
19–44	777	362	250	46 (18.4)	86 (67–110)		1,754	1,596	1,171	174 (14.9)	135 (118–153)	1.6 (1.2–2.1)#
45–59	215	135	93	9 (9.7)	61 (32–104)		493	556	413	56 (13.6)	152 (120–191)	2.5 (1.3–4.9)#
>60	128	84	59	1 (1.7)	8 (0.2–44.0)		294	362	275	15 (5.5)	68 (42–105)	8.7 (1.4–360.0)#
Influenza B												
0–5	230	499	411	12 (2.9)	65 (37–108)		498	1,174	1,036	103 (10.0)	235 (194–281)	3.6 (2.1–6.6)
6–18	486	435	281	15 (5.3)	47 (30–71)		1,061	1,245	1,012	138 (13.6)	160 (137–186)	3.4 (2.2–5.5)
19–44	777	362	250	7 (2.8)	13 (6–24)		1,754	1,596	1,171	100 (8.5)	77 (65–92)	6.0 (3.2–12.8)
45–59	215	135	93	0	0 (0–17)		493	556	413	19 (4.6)	53 (34–77)	0 (0–2.9)**
>60	128	84	59	0	0 (0–29)		294	362	275	17 (6.2)	75 (47–113)	0 (0–2.4)**

*ARI, acute respiratory infection. †For surveillance. ‡For influenza. §Per 1,000 person-years. ¶p<0.0001 (incidence lower in postpandemic than in pandemic period). #p<0.02 (incidence higher in postpandemic than in pandemic period). **No influenza B–positive results for these age groups in pandemic period.

The overall IR for pH1N1 was higher for children <18 years of age (345) than for adults >18 years of age (69) during the pandemic period, whereas IRs were similar among children (199) and adults (131) during the postpandemic period. However, the IR of pH1N1 was significantly higher (p<0.0001) among children during the pandemic period compared with the postpandemic period (IRR 0.6), whereas the rate for adults was higher during the postpandemic period (IRR 1.8) (Figure 2, insets). In contrast, the IR for influenza B remained 2.5× higher for children (IR 184) than adults (IR 72) during the postpandemic period.

## Conclusions

Data from this large-scale, community-based, prospective surveillance program demonstrated that the introduction of the pH1N1 strain into a naive population in northern India initially affected preschool- and school-aged children during the first phase of the pandemic, with a demographic shift to adults during the postpandemic phase. The analysis has several unique characteristics. By chance, the study began soon after the emergence of pandemic influenza in northern India, which enabled the analysis of pandemic and postpandemic periods in the same study population; we were able to measure incidence of pandemic and influenza B cocirculating in the community. Because we used active surveillance, we likely captured most febrile ARI cases among all age groups, and therefore our results likely are robust and unbiased. If similar patterns occurred during future pandemics in other (e.g., urban) populations, interventions should be redirected from children to adults during the postpandemic phases.

School-aged children often are at the leading edge of a pandemic, and they remained the top-priority group for vaccination during the 2009 influenza pandemic (*11*,*12*). Our findings suggest that the high IR of pH1N1 among schoolchildren led to naturally acquired immunity, which lowered the susceptibility of this population to illness during the postpandemic phase. Conversely, an increase in IR among adults during the postpandemic phase supports previous observations that pandemic influenza transmission shifts from highly susceptible children during a pandemic period to adults during the postpandemic phase (*9*). These age-specific demographic shifts in IRs were also observed for the major pandemics of 1918, 1957, and 1968 (*5*–*7*).

Our study has several limitations. First, the population under surveillance during the pandemic was relatively small because of phased enrollment during the initial study implementation and not all febrile ARI case-patients could be sampled; these effects were corrected by using the person-time method for calculating IRs. However, we recognize that febrile ARI case-patients who were not sampled may have had milder disease, and, therefore, influenza rates may vary. Second, the IRs of pH1N1 reported during the pandemic period may be underestimates because the initial pandemic peak in nearby areas was observed during August 2009, with highest positivity rates for those 6–18 years old (*11*). Third, while no routine influenza vaccination program exists in this community, the incidence rates for pH1N1 among children may have been skewed because of protection afforded by seasonal influenza trivalent vaccination, administered during November 2009 (*13*–*15*). However, the effect, if any, of this vaccination on influenza B incidence has not been determined.

Despite these limitations, we believe that the age-specific demographic shift we observed for the 2009 influenza pandemic will be useful for future modeling projects addressing this issue. Future pandemic preparedness activities should focus on targeted interventions for different age groups as the pandemic evolves, as well as on the severity of disease in different age groups.
